# Ocular disconjugacy cannot be measured without establishing a solid spatial reference

**DOI:** 10.12688/f1000research.6162.2

**Published:** 2015-04-22

**Authors:** Jun Maruta

**Affiliations:** 1 Brain Trauma Foundation, One Broadway, 6th Floor, New York, NY 10007, USA

**Keywords:** Mild traumatic brain injury, mTBI screening, vergence

## Abstract

This correspondence points out a need for clarification concerning the methodology utilized in the study “Eye tracking detects disconjugate eye movements associated with structural traumatic brain injury and concussion”, recently published in *Journal of Neurotrauma.* The authors of the paper state that binocular eye movements were recorded using a single-camera video-oculography technique and that binocular disconjugate characteristics were analyzed without calibration of eye orientation. It is claimed that a variance-based disconjugacy metric was found to be sensitive to the severity of a concussive brain injury and to the status of recovery after the original injury. However, the reproducibility of the paper’s findings may be challenged simply by the paucity of details in the methodological description. More importantly, from the information supplied or cited in the paper, it is difficult to evaluate the validity of the potentially interesting conclusions of the paper.

## Correspondence

I wish to point out a need for clarification concerning the methodology utilized in the study “Eye tracking detects disconjugate eye movements associated with structural traumatic brain injury and concussion” by Samadani *et al.*, 2015 ^1^. The authors state that binocular eye movements were recorded using a single-camera infrared-based video-oculography technique (EyeLink 1000, SR Research, Ontario, Canada) and that binocular disconjugate characteristics were analyzed without calibration of eye orientation. The authors claim that their variance-based disconjugacy metric was sensitive to the severity of a concussive brain injury and to the status of recovery after the original injury.

The EyeLink 1000 system is an excellent eye tracker with a single high resolution camera and an infrared light source affixed to a side of the camera. This system uses a dark pupil-corneal reflection principle for tracking eye movements. A typical recording setup consists of a computer monitor with which the visual stimuli are presented to the subject and the camera unit (placed in front of the monitor base) with which the eye movement is recorded monocularly or binocularly. The system has an option of easily outputting image-based, uncalibrated eye coordinates with hundreds of units representing 1° of eye rotation.

The concern I would raise with the Samadani *et al.* paper is the unclearness of the relationship between their metric and binocular disconjugacy. Logically, for an identical amount of eye rotation, any asymmetry in the spatial relationship that the camera or the infrared light source has with the two fellow eyes would result in different extents of relocation of the images of the pupils or corneal reflections. Asymmetries exist because there is a physical separation between the two eyes as well as between the camera and the infrared light source. For instance, the camera may be centered in front of the subject to obtain a symmetric view of the pupils for a gaze along the midline, but the infrared light source cannot be centered simultaneously, the consequence of which is an asymmetric view of the corneal reflections.

The individually variable physical separation of the two eyes contributes to variations in the extent of asymmetry in the camera view of the landmark features of the eyes. It may be asserted that the group-wise comparisons utilized by Samadani *et al.* are robust to effects of subject-wise asymmetry and the equally constant lack of calibration. However, a systematic between-group bias may be created when the demographic composition of the subject groups are different. For example, having a larger male-to-female ratio in one group could increase the extent of binocular asymmetry in uncalibrated data since men tend to have a larger interocular distance ^2, 3^. Incidentally, Samadani *et al.* note a tendency toward the positive head CT group having more males than the non-injured control group, with the positive head CT group of 13 patients being 35.9% female and the control group of 64 subjects being 47.9% female. (Curiously, the percentage of female subjects times the group size does not yield a whole number in any of the four subject groups in the Samadani *et al.* paper.)

There are still other variables that confound the relationship between eye rotation and changes in pixel coordinates. Although the biometric characteristics of eyes are highly symmetrical within individuals, they are not perfectly symmetrical ^4^ and a 1–2% non-conformity in corneal curvature or axial length is not uncommon. Each of the two fellow eyes has its own function that maps pixel movement to the eye rotation, and this mapping is not linear. Thus, the arithmetic difference between the uncalibrated coordinates of the two eyes is quite removed from a physical representation of gaze misalignment.

Beyond the factors associated with the raw data, the analytic methods in the paper also do not seem to be constructed with a clear intent. It is puzzling why the disconjugacy metric is represented by the variance of the left-right differences after independently averaging for each eye the uncalibrated coordinates over several cycles for a given stimulus position, as opposed to the straightforward variance of the left-right differences at all sample points. Furthermore, the ranges of outcome values presented in the series of figures run from 0 to at most 0.25, but how the value 0 could have been obtained is not clear. The question arises because in the two eyes’ uncalibrated coordinates there must be a constant bias related to the interocular distance. Lastly, what the high end of the outcome range represents is not clear. Since one unit in EyeLink’s uncalibrated data output is smaller than 0.01° of eye rotation, being able to report differences in 0.25 square units or less seems implausible. If the raw data were numerically centered or scaled, the procedure should have been noted in the text.

Since disconjugacy is spatial in nature, a solid reference is needed. The authors discuss some valid points regarding potential pitfalls associated with calibration and phoria. However, these points can be directly addressed by implementing a calibration procedure under monocular viewing ^5, 6^. A comparison between the results from thus calibrated and uncalibrated data, and a demonstration of test-retest reliability could have improved the paper.

In summary, the reproducibility of the paper’s findings may be challenged simply by the paucity of details in the methodological description. More importantly, however, from the information supplied or cited in the paper it is difficult to evaluate the validity of the potentially interesting conclusion that deficits in conjugacy of eye movements may quantitate physiologic impact of brain injury.
