# Supplementary material for: Ocular disconjugacy cannot be measured without establishing a solid spatial reference
Source: F1000Res. 2015 Apr 22;4:71. Originally published 2015 Mar 17. [Version 2] doi: 10.12688/f1000research.6162.2 (PMC4448744; doi:10.12688/f1000research.6162.2)
Supplement: Peer review history

# Peer review: Referee response for version 2

**DOI:** 10.5256/f1000research.6868.r8688

**Published:** 2015-05-15

**Authors:** Marcus Nyström

**Affiliations:** 1 Humanities Laboratory, Lund University, Lund, Sweden

I agree with the methodological aspects that the author highlights in his correspondence, and that the Samadani *et al. *paper would have been improved by a more detailed description of their methods as well as a validation or their method to compute disconjugacy.

That said, it is uncertain to what extent the methodological aspects influence the differences between the experimental groups found by Samadani *et al. *The correspondence would therefore benefit from a quantitative investigation addressing the practical significance of (at least some of) the issues raised.

I have read this submission. I believe that I have an appropriate level of expertise to confirm that it is of an acceptable scientific standard, however I have significant reservations, as outlined above.

# Peer review: Referee response for version 2

**DOI:** 10.5256/f1000research.6868.r8471

**Published:** 2015-04-24

**Authors:** Christopher Tyler

**Affiliations:** 1 Brain Imaging Center, Smith-Kettlewell Eye Research Institute, San Francisco, CA, USA

The title of the piece is not logically correct, and needs further modification. Although the author’s clarification explains how the asymmetry of the light source could, in principle lead to discrepancies in the measurement of ocular disconjugacy, it is based on a long chain of reasoning that is far from establishing the claim of the title, that “Ocular disconjugacy cannot be measured without establishing a solid spatial reference.”  What the Correspondence establishes is that ocular disconjugacy measures could, in principle, be biased by asymmetries in the geometry of eye tracking camera illumination. To my mind, however, he has not yet established that the ocular disconjugacy measures were in fact biased in this way. He admits that, if the male-female ratios were equated, there would be no basis for a bias between the TBI and the control groups, even given the asymmetry of the illumination. Thus, even if the present result were significantly biased by the small differences in the male-female ratios of the present study, it would still not be the case that “ocular disconjugacy cannot be measured without establishing a solid spatial reference,”  since the any bias introduced by interpupillary differences could be compensated by matching the interpupilllary distances of the controls to those of the test subjects.

The added phrase should be “affixed to one side of the camera”. The sentence “Asymmetries exist because there is a physical separation between the two eyes as well as between the camera and the infrared light source.” is logically incorrect. Asymmetries exist because the light source is asymmetrically placed with respect to the symmetry axis of the two eyes.  If the displaced infrared source were either above or below the camera lens, i.e. if it were on the symmetry axis, there would be no asymmetry in the view of the eyes (assuming that the eyes themselves were symmetric). Of course, the first half of the sentence is definitely not true on its own: “asymmetries exist because there is a physical separation between the two eyes”.

The concept of a bias introduced by consistent male-to-female ratio differences developed in the response goes some way to addressing the issue of bias introduced by the asymmetry, but it has to be established for the groups in the study in order to be a relevant criticism. The male-to-female ratio differences between groups were small, and were not statistically significant for these small groups.  Can the author show that these small ratio differences were quantitatively sufficient to account for the Samadani *et al.* results (in terms of the reported range of male-female interpupillary distances in the subject populations that they used)?

I have read this submission. I believe that I have an appropriate level of expertise to confirm that it is of an acceptable scientific standard, however I have significant reservations, as outlined above.

# Response

**Published:** 2015-04-29

**Authors:** Jun Maruta

**Affiliations:** Brain Trauma Foundation, USA

It seems that this Correspondence is somehow viewed as an attempt to explain the findings of Samadani *et al*. This responsibility rests with Samadani *et al*. In my view, that ocular disconjugacy cannot be measured without a solid spatial reference is so by virtue of what defines ocular disconjugacy, rather than needing to be established. In any individual measurement, non-pathologically-related asymmetries exist between the uncalibrated coordinates of the two eyes; thus, their arithmetic difference does not represent gaze misalignment. The physical meanings of the metrics that Samadani *et al*. claim to be associated with TBI are not clear, and the reported values associated with these metrics do not follow the explanation provided in the text. It is possible that some output of a magical black box should indeed be found to be associated with TBI. However, my argument would still be, “What is the science behind it?”

Regarding the sentence “Asymmetries exist because there is a physical separation between the two eyes as well as between the camera and the infrared light source,” it is made clear that, in the context, the relative displacement of the infrared light source from the camera is in the lateral direction. The first half of the sentence is not meant to stand alone.

# Peer review: Referee response for version 1

**DOI:** 10.5256/f1000research.6606.r8105

**Published:** 2015-04-08

**Authors:** Christopher Tyler

**Affiliations:** 1 Brain Imaging Center, Smith-Kettlewell Eye Research Institute, San Francisco, CA, USA

Overall, this Correspondence does not offer a significant criticism of the paper of Samadani *et al.* (2015). The author does not specify what kinds of asymmetries exist due to the camera/source geometry. Usually the subject is centered in the Eyelink viewing aperture, so the view of the eyes would be symmetric. The attempt to argue that a 1-2% asymmetry between the eyes would significantly degrade the assessment capability does not seem plausible.

The criticism of methodological unclarity may be justified in itself, but it has no bearing on the outcome of the paper since all patients would be equally subject to the same degree by the effects of asymmetry and lack of calibration. As stated, none of the criticisms suggest a systematic bias between the different patient categories. The significant differences among categories cannot therefore be attributed to any of the factors raised by the author, and controlling these factors should only improve the significance of the Samadani *et al.* results.  The author’s challenge to the findings of the paper is thus easily refuted.

Moreover, despite the fact that this Correspondence is entirely concerned with calibration, it neglects to mention the fact that a core motivation for the Samadani *et al.* paper is the issue of a misleading spatial calibration metric. They are making the point that restrictions of eye movement may restrict the array of calibration positions, providing a false assessment that the subsequent test movements are normal. Their goal is thus to use the *uncalibrated* differences between the two eyes (which they term “temporal calibration”) as a probe for deficits that would be masked by the spatial calibration procedure. It is this strategy that the author should have addressed directly.

On a stylistic note, I do not favor using the English possessive “s” on the Latin phrase “et al(ia)” and recommend the form above, as in “the Samadani *et al*. paper”.

I have read this submission. I believe that I have an appropriate level of expertise to state that I do not consider it to be of an acceptable scientific standard, for reasons outlined above.

# Response

**Published:** 2015-04-10

**Authors:** Jun Maruta

**Affiliations:** Brain Trauma Foundation, USA

I thank Dr. Tyler for his comments and the opportunity to think through what I had written again. One revision I can make in the second paragraph of the original text is to add “a side of” to “an infrared light source affixed to the camera” so that the phrase reads “an infrared light source affixed to a side of the camera.” This revision would make it clearer that the camera may be centered in front of the subject but the infrared light source cannot be centered simultaneously, the consequence of which is an asymmetric view of the corneal reflections. I can also specify that EyeLink 1000 uses a dark pupil-corneal reflection principle for tracking eye movements.

Another clarification I can offer is to point out that the physical separation of the two eyes is individually variable [1,2]. This variability contributes to variations in the extent of asymmetry in the camera view of the landmark features of the eyes. With this consideration, Dr. Tyler’s assertion, that all subjects would be equally subject to the same degree by the effects of asymmetry, may be only partially correct. A systematic bias may indeed be created when the demographic composition of the subject groups are different. For example, having a larger male-to-female ratio in one group could increase the extent of binocular asymmetry in uncalibrated data since men tend to have a larger interpupillary distance [1,2]. Incidentally, Samadani et al. note a tendency toward the positive head CT group having more males than the non-injured control, with the positive head CT group of 13 patients being 35.9% female and the control group of 64 subjects being 47.9% female. (Curiously, the percentage of female subjects times the group size does not yield a whole number in any of the four subject groups in the Samadani et al. paper.)

The assessment capability claimed by Samadani et al. appears to be variability of binocular alignment on the order of 0.01° magnitude. This value seems too small in light of the two sources of asymmetries discussed above in uncalibrated gaze-associated binocular measures, and also in light of 1-2% asymmetry between the two eyes.

Regarding the problems that Samadani et al. point out in terms of spatial calibration, my intent is not to dispute the existence of alternatives to eye movement assessments based on spatial calibration. Rather, my emphasis is that disconjugacy itself is spatial in nature, and thus its quantification requires a solid spatial reference frame. This intent may be made clearer by including the term “spatial” in the title so that it reads “Ocular disconjugacy cannot be measured without establishing a solid spatial reference.”

Finally, in my future writing I will keep in mind your recommendation regarding mixing the English possessive on the Latin phrase “et al.”

References:

1. Pryor HB: Objective measurement of interpupillary distance. Pediatrics. 1969; 44(6): 973-7.

2. Gordon CC, Churchill T, Clauser CE, et al.: 1988 anthropometric survey of U.S. Army personnel: methods and summary statistics. U.S. Army Natick Research, Development and Engineering Center, Natick MA. 1989.

# Peer review: Referee response for version 1

**DOI:** 10.5256/f1000research.6606.r7999

**Published:** 2015-03-31

**Authors:** Johannes van der Steen

**Affiliations:** 1 Department of Neuroscience, Erasmus Medical Center, Rotterdam, Netherlands

The author makes a justified appeal for clarification of methodological issues concerning the study “Eye tracking detects disconjugate eye movements associated with structural traumatic brain injury and concussion” by Samadani *et al.*, 2015. The arguments raised by J. Maruta are very valid. Apart from clarification there is also a need for validation before this technique can be introduced in e.g. emergency rooms to quantify the severity of a concussive brain injury and the degree of recovery.

I have read this submission. I believe that I have an appropriate level of expertise to confirm that it is of an acceptable scientific standard.
